# Maternal Smoking during Pregnancy and the Prevalence of Autism Spectrum Disorders, Using Data from the Autism and Developmental Disabilities Monitoring Network

**DOI:** 10.1289/ehp.1104556

**Published:** 2012-04-25

**Authors:** Amy E. Kalkbrenner, Joe M. Braun, Maureen S. Durkin, Matthew J. Maenner, Christopher Cunniff, Li-Ching Lee, Sydney Pettygrove, Joyce S. Nicholas, Julie L. Daniels

**Affiliations:** 1 Zilber School of Public Health, University of Wisconsin–Milwaukee, Milwaukee, Wisconsin, USA; 2 Department of Environmental Health, Harvard School of Public Health, Boston, Massachusetts, USA; 3 Department of Population Health Sciences, University of Wisconsin School of Medicine and Public Health, Madison, Wisconsin, USA; 4 Waisman Center, University of Wisconsin, Madison, Wisconsin, USA; 5 Department of Pediatrics, University of Arizona College of Medicine, Tucson, Arizona, USA; 6 Department of Epidemiology, Johns Hopkins Bloomberg School of Public Health, Baltimore, Maryland, USA; 7 College of Public Health, University of Arizona, Tucson, Arizona, USA; 8 Department of Medicine, Division of Biostatistics and Epidemiology, Medical University of South Carolina, Charleston, South Carolina, USA; 9 Department of Epidemiology, University of North Carolina at Chapel Hill, Chapel Hill, North Carolina, USA

**Keywords:** autism, epidemiology, intellectual disability, surveillance, tobacco

## Abstract

Background: Reported associations between gestational tobacco exposure and autism spectrum disorders (ASDs) have been inconsistent.

Objective: We estimated the association between maternal smoking during pregnancy and ASDs among children 8 years of age.

Methods: This population-based case–cohort study included 633,989 children, identified using publicly available birth certificate data, born in 1992, 1994, 1996, and 1998 from parts of 11 U.S. states subsequently under ASD surveillance. Of these children, 3,315 were identified as having an ASD by the active, records-based surveillance of the Autism and Developmental Disabilities Monitoring Network. We estimated prevalence ratios (PRs) of maternal smoking from birth certificate report and ASDs using logistic regression, adjusting for maternal education, race/ethnicity, marital status, and maternal age; separately examining higher- and lower-functioning case subgroups; and correcting for assumed under-ascertainment of autism by level of maternal education.

Results: About 13% of the source population and 11% of children with an ASD had a report of maternal smoking in pregnancy: adjusted PR (95% confidence interval) of 0.90 (0.80, 1.01). The association for the case subgroup autistic disorder (1,310 cases) was similar: 0.88 (0.72, 1.08), whereas that for ASD not otherwise specified (ASD-NOS) (375 cases) was positive, albeit including the null: 1.26 (0.91, 1.75). Unadjusted associations corrected for assumed under-ascertainment were 1.06 (0.98, 1.14) for all ASDs, 1.12 (0.97, 1.30) for autistic disorder, and 1.63 (1.30, 2.04) for ASD-NOS.

Conclusions: After accounting for the potential of under-ascertainment bias, we found a null association between maternal smoking in pregnancy and ASDs, generally. The possibility of an association with a higher-functioning ASD subgroup was suggested, and warrants further study.

Autism is estimated to occur in 1 of 88 children [Centers for Disease Control and Prevention (CDC) 2012]. Although the etiology of autism is still unknown, both genetic factors and environmental exposures have been implicated (Newschaffer et al. 2007). Exposure to exogenous agents may especially affect autism risk when it occurs during fetal development (Rodier et al. 1996).

*In utero* tobacco exposure via direct smoking by the mother has been associated with neurodevelopmental deficits such as cognitive impairments and behavioral problems, suggesting that such exposure is neurotoxic (Braun et al. 2009; DiFranza et al. 2004; Mendola et al. 2002). Potential mechanisms that may underlie this relationship include fetal hypoxia and modulation of neurotransmitter systems via nicotinic acetylcholine receptors (Burstyn et al. 2011; Slotkin 2004; Soothill et al. 1996). Yet primary tobacco exposure in pregnancy remains an important and preventable public health concern: 13% of infants in the United States were exposed to maternal smoking during pregnancy in 1999–2006 (Dietz et al. 2011).

The literature on tobacco exposure during pregnancy and subsequent autism spectrum disorders (ASDs) is inconclusive. Studies in the United States, Europe, and China have reported associations that are inverse (Juul-Dam et al. 2001; Williams et al. 2003), close to null (Burstyn et al. 2010; Larsson et al. 2005; Lee et al. 2011; Maimburg and Vaeth 2006) and elevated (Hultman et al. 2002; Indredavik et al. 2007; Larsson et al. 2009; Zhang et al. 2010). These studies varied widely in design, case definition, and ability to control for social class influences. Furthermore, the practices of autism screening, access to diagnostic health services, and social norms around tobacco use varied greatly among the studied countries. Such factors may have influenced study results.

To clarify the association between maternal smoking in pregnancy and the subsequent development of an ASD, we conducted a study using a large number of cases from a population-based U.S. surveillance program, the Autism and Developmental Disabilities Monitoring (ADDM) network. We controlled for social and demographic confounding factors, evaluated case subgroups, and explored data limitations using sensitivity analyses.

## Methods

*Study population.* We implemented a population-based, case–cohort design, using ASD surveillance data from ADDM and publicly available birth certificate files from the National Center for Health Statistics (NCHS) (CDC 2009a) and the North Carolina State Center for Health Statistics (Howard W. Odum Institute for Research in Social Science at the University of North Carolina at Chapel Hill 2009). Our study population was defined as all children born in 1992, 1994, 1996, and 1998 who resided at birth within regions subsequently under ADDM surveillance during their 8th year of life. We excluded some counties with populations < 100,000 in which birth county is suppressed in birth certificate files and excluded some ADDM regions in which surveillance was incomplete within county boundaries. Finally, we restricted the sample to ADDM sites that were able to successfully obtain the needed birth certificate variables. Regions included for at least 1 year in this study included 5 northern counties in Alabama, all of Arkansas, Miami-Dade county in Florida, 5 counties in metropolitan Atlanta in Georgia, Baltimore county and 5 surrounding counties in Maryland, 6 counties in metropolitan St. Louis in Missouri and Illinois, Union County just south of Newark in New Jersey, 10 counties surrounding Greensboro and Durham in North Carolina, Philadelphia County in Pennsylvania, 5 counties in southeastern Wisconsin including Milwaukee, and all of West Virginia.

*Surveillance ascertainment and case subgroups.* Cases were all children with ADDM surveillance-ascertained ASDs born within the source population as defined above. The ADDM network has performed active, population-based surveillance for ASDs in select regions of the United States biannually since 2000. The surveillance methodology does not directly evaluate children, but relies on developmental records through the child’s 8th year of life at key agencies, including medical agencies, early intervention services, and public schools (CDC 2007, 2009b; Van Naarden Braun et al. 2007). An ADDM clinician ascertained whether characteristics and behaviors in a child’s developmental record met the standardized ADDM case definition for ASDs, based on the *Diagnostic and Statistical Manual, Fourth Edition–Text Revision* (DSM-IV-TR) (American Psychiatric Association 2000). Information on these children was obtained in compliance with all applicable regulations for the protection of human health and educational data, including approval by institutional review boards in each ADDM region.

The ADDM network recorded variables that further characterized the phenotype for children meeting the ADDM case definition of ASD. Variables that were abstracted directly from the developmental record included *a*) community diagnosis: whether a community provider had ever diagnosed the child with autistic disorder (AD) and/or ASD not otherwise specified (ASD-NOS); and, if so, *b*) timing of diagnosis: whether the child’s age in months at the earliest documented diagnosis was early (dichotomized using the median value, < 50 months) or late (≥ 50 months), and *c*) co-occurring intellectual disability (ID), defined as IQ ≤ 70 on tests such as the Battelle–cognitive domain (Newborg 2004), Differential Ability Scales (Elliott 2007), Stanford-Binet–4th ed. (Thorndike et al. 1986), Wechsler Intelligence Scale for Children-III (Wechsler 1991), and the Wechsler Preschool and Primary Scale of Intelligence (Wechsler 1989). A fourth case subgrouping variable was newly derived by ADDM clinicians based on a review of the entire composite record; clinicians classified cases as AD, requiring documented symptoms corresponding to DSM-IV-TR criteria for AD, or ASD-NOS, requiring fewer or less severe symptoms and including Asperger’s disorder and pervasive developmental disorder.

Children could be identified as cases only if they resided within the surveillance regions when they were 8 years of age. We limited the case group to those children also born within the surveillance areas so that our case group arose from the underlying population. We determined county of birth from birth certificate data obtained by each ADDM surveillance site.

*Maternal smoking in pregnancy and covariates.* Information on maternal smoking during pregnancy was obtained from birth certificate data. Smoking is collected using a yes/no check-box in a method that varies by state but usually involves abstraction of the medical record. Demographic factors, including maternal education, age, marital status, and race/ethnicity were also obtained from birth certificates. We used a variable of county population size available in the NCHS birth certificate data as a proxy for the urbanicity of each county (Table 1).

**Table 1:** Prevalence of ASDs and case subgroups by child and family characteristics, with characteristics of the source population

Prevalence per 1,000 for case groups									Main source population (n = 633,989)				
Characteristic	All ASD (n = 3,315)	ADa (n = 1,310)	ASD-NOSa (n = 375)	ASD with IDb (n = 584)	ASD without IDb (n = 754)	n	Percent by characteristic	Percent smoking in pregnancy
Overall		5.2		4.4		1.3		2.2		2.9						
Exposure																
Smoking in pregnancy																
Yes		4.4		3.7		1.5		1.7		2.7		83,883		13		
No		5.3		4.5		1.2		2.3		2.9		550,106		87		
Child Characteristics																
Sex																
Female		2.0		1.6		0.4		1.0		0.9		309,861		49		13
Male		8.2		7.0		2.1		3.4		4.8		324,128		51		13
Race/Ethnicity																
Non-Hispanic white		5.8		5.3		1.6		1.7		3.6		385,493		61		16
Non-Hispanic black		4.5		3.6		0.9		3.1		1.9		186,113		29		10
Hispanic		3.4		2.7		0.7		1.6		1.7		45,541		7		4
Other		4.3		3.0		0.7		1.7		2.5		16,842		3		4
Family and county characteristics																
Maternal education																
< High school		3.1		2.4		0.8		2.0		1.4		117,300		19		26
High school degree		4.4		3.6		1.1		2.4		2.0		204,026		32		18
Some college		6.0		5.4		1.3		2.6		3.4		139,069		22		10
College degree		6.8		5.7		1.7		1.9		4.4		173,594		27		2
Maternal age (years)																
10–19		2.6		1.9		0.8		1.7		1.2		81,090		13		16
20–24		4.0		3.3		0.9		1.9		1.8		147,507		23		17
25–29		5.3		4.1		1.3		2.2		2.9		173,152		27		12
30–34		6.4		5.6		1.5		2.5		3.6		154,651		24		11
35–39		7.6		6.6		1.7		3.0		4.9		66,354		10		11
40–53		8.4		7.1		1.7		2.2		7.0		11,235		2		10
Married																
Yes		5.9		5.0		1.4		2.2		3.4		417,082		66		10
No		3.9		3.2		1.0		2.3		1.7		216,907		34		20
County population size																
> 1,000,000		3.4		2.7		0.8		NI		NI		52,236		8		8
500,000 – 1,000,000		5.5		4.3		1.6		2.3		2.2		216,687		34		11
250,000 – 500,000		5.9		5.1		1.2		2.2		3.6		160,247		25		12
100,000 – 250,000		5.0		5.0		1.2		2.4		2.5		131,894		21		16
< 100,000		4.5		4.4		1.3		1.9		2.9		72,925		12		24
Study design characteristics																
Birth/surveillance year																
1992/2000		4.4		3.3		1.5		1.8		2.0		65,899		10		15
1994/2002		4.5		NI		NI		2.0		2.6		231,544		37		16
1996/2004		5.7		4.1		1.0		2.3		2.9		120,301		19		12
1998/2006		5.9		4.7		1.3		2.7		3.8		216,245		34		11
Site																
AL		4.3		3.9		0.9		2.4		1.1		45,851		7		11
AR		5.0		NI		NI		2.0		2.9		34,311		5		20
FL		2.7		2.0		0.7		NI		NI		31,511		5		2
GA		5.3		4.4		1.0		2.1		2.9		159,493		25		7
MD		5.1		3.3		2.4		NI		NI		70,698		12		13
MO		6.8		7.3		2.5		NI		NI		84,359		13		16
NC		6.0		5.9		0.9		2.7		3.2		59,005		9		16
NJ		7.9		4.8		3.6		NI		NI		14,291		2		7
PA		4.5		3.5		0.9		NI		NI		20,725		3		16
WI		4.7		4.1		1.2		NI		NI		70,912		11		17
WV		4.3		NI		NI		NI		NI		42,833		7		27
Abbreviations: AD, autistic disorder; ASD, autism spectrum disorders; ASD-NOS, autistic spectrum disorder–not otherwise specified; ASD with ID, autism spectrum disorder with co-occurring intellectual disability; ASD without ID, autism spectrum disorder without co-occurring intellectual disability; NI, not included. aAD and ASD-NOS subgroups refer to subgroups determined by ADDM clinicians. Subsets of ADDM data had available information on ADDM-determined AD and ASD-NOS. bAnother data subset had at least 80% complete data on co-occurring ID.																

*Primary statistical analysis.* We estimated prevalence ratios (PRs) of ASD by level of maternal smoking (yes/no) using logistic regression. We did not identify or remove cases from the denominator data; consequently, cases were included in the denominator data set representing all children born in the eligible geographic regions and birth years. Thus, odds ratios from these models are mathematically equivalent to PRs. We were unable to confirm the ASD status for individuals who moved out of the surveillance region between birth and 8 years of age, leading to a slight underestimation of prevalence. We included factors in multivariable models that may have the potential to confound the association between maternal smoking, and excluded factors that may be acting as causal intermediaries because they are influenced by maternal smoking (e.g., low birth weight) (Cole and Hernan 2002; Greenland and Brumback 2002). Selected potential confounders included maternal education (modeled using restricted quadratic splines) (Durkin et al. 2010), race and ethnicity (categorized as non-Hispanic white, non-Hispanic black, Hispanic, or other) (Mandell et al. 2009), marital status (yes/no), and maternal age (restricted quadratic splines) (Durkin et al. 2008). Next, we evaluated whether county population size (in five categories as in NCHS data), birth year (as categories), and surveillance site (as categories) confounded our estimates.

We evaluated modification of the association between maternal smoking and ASD by *a*) child sex, because it has been found to modify other environmental–chemical–neurodevelopmental associations (Bellinger et al. 1990; Braun et al. 2011; Ris et al. 2004); *b*) maternal race/ethnicity and *c*) education; and variables that may capture differences in ADDM surveillance activities or general temporal or spatial trends: *d*) birth year and *e*) county population size. Modifiers were evaluated on the multiplicative scale by inspecting PRs stratified by the potential modifier and by performing likelihood ratio tests. The likelihood ratio tests compared a fully adjusted model to a model that additionally included cross-product terms between a potential modifier and maternal smoking. Factors for which the likelihood ratio test *p-*value was < 0.10 were considered to modify the association between maternal smoking and ASDs.

We repeated our multivariable models for several case subgroups in exploratory analyses, assuming that different subgroups may exhibit differential susceptibility to tobacco smoke. Subgroups that corresponded to higher- and lower-functioning ASDs, such as subgroups based on co-occurring intellectual disabilities (ID), have been suggested as ASD endophenotypes that correspond to genetic liability (Szatmari et al. 2007). We used the following available variables from ADDM to define case subgroups: whether a prior community diagnosis was AD or ASD-NOS, the timing of first diagnosis (assuming that earlier diagnosis in part served as a marker of more numerous or more severe symptoms), ADDM-determined subgroup (AD or ASD-NOS), and the presence of co-occurring ID. Because of differences in the data available between sites and years, some analyses of ASD subgroups were limited to a data subset that contained the needed variables (Table 2).

**Table 2:** Distribution of maternal smoking during pregnancy for ASDs and case subgroups

Group	Case subgroup	No. of cases	Percent smoking in pregnancy
Main source population						
Births				633,989		13.2
ADDM-ascertained ASD				3,315		11.2
Community provider diagnosesa		Any AD		949		9.9
		ASD and no AD		870		11.2
Timing of first community diagnosisa		< 50 months		994		9.3
		≥ 50 months		1,108		12.2
Source population with ADDM-determined subgroup						
Births				297,493		10.7
ADDM-ascertained ASD				1,685		9.9
ADDM–designated subgroup		AD		1,310		9.0
		ASD-NOS		375		13.1
Source population with ID data						
Births				261,786		11.1
ADDM-ascertained ASD				1,409		9.3
Co-occurring IDa		Yes		584		8.4
		No		754		10.3
Abbreviations: AD, autistic disorder; ADDM, Autism and Developmental Disabilities Monitoring Network; ASD, autism spectrum disorders; ASD-NOS, autistic spectrum disorder–not otherwise specified; ID, intellectual disability. aThese outcome subgroups do not add up to the total number of children with an ASD because some children were missing data needed to place them in a subgroup.						

*Sensitivity analyses.* Autism has consistently been found to be more prevalent in groups of higher social class in the United States, leading to concerns that autism may be under-ascertained in children of lower social class. Such gradients are even found in ADDM data, despite its active surveillance methodology that can recognize a case without a prior documented diagnosis (Durkin et al. 2010). To evaluate the impact of under-ascertainment on our results, we performed a sensitivity analysis correcting for such outcome misclassification. Because of the strong association between maternal education and smoking in pregnancy (CDC 2010), ascertainment that varies by maternal education has the potential to affect results, even without an assumption of differential ASD ascertainment within smoking strata. We used standard formulas to correct for outcome misclassification and varied the specificity assumptions as allowable without creating negative cell counts (Rothman et al. 2008). We adjusted the number of cases using different estimates of sensitivity in each stratum of maternal education, assuming the highest outcome sensitivity in the stratum with a college degree and comparatively less sensitivity for all other educational strata based on the ASD prevalence observed in our data [see Supplemental Material, Table S1(http://ox.doi.org/10.1289/ehp.1104556)]. This process assumed that only the surveillance ascertainment of ASD, but not the true prevalence of the condition, varied by maternal education. We constrained our stratum-specific sensitivity values so that the overall outcome sensitivity corresponded to that found in an ADDM evaluation study: 0.60 (Avchen et al. 2010). Outcome sensitivity values for ASD by strata of maternal education were as follows: college degree: 0.80; some college: 0.72; high school degree: 0.53; and less than high school: 0.35 (see Supplemental Material, Tables S1 and S2). We also performed outcome misclassification corrections separately for ADDM-determined AD and ASD-NOS (see Supplemental Material, Tables S1 and S3). After adjusting numbers of cases and controls using an Excel spreadsheet, we calculated confidence intervals (CIs) using PROC Freq/CMH in SAS (SAS Institute Inc., Cary, NC) applied to the resultant simulated data (Robins et al. 1986).

To evaluate a potential selection bias from including infants who died in the first year of life, we performed an analysis removing infant deaths in regions and years for which information on infant death was available, using the Birth Cohort Linked Birth–Infant Death Data Files from NCHS (CDC 2009a). These files were available for birth years 1996 and 1998 in counties with populations > 250,000.

We performed a subanalysis to evaluate the impact of residential mobility on our results, because the source population included children who had moved out of the study area and could not be identified as ASD cases at age 8. This subanalysis was limited to children from North Carolina born in 1994 and 1996. We traced the residential histories of a random sample of this birth cohort to determine residency within the surveillance catchment area at age 8. Tracing was conducted by searching on maternal and paternal names from the birth certificate using commercial databases of multiple residences over time provided by LexisNexis. We then compared PRs of smoking and ASD using *a*) a denominator of all included North Carolina children versus *b*) a denominator of children remaining within the North Carolina surveillance area.

## Results

A total of 633,989 births met our inclusion criteria by residency and year of birth and had complete information on relevant covariates. Of these, 3,315 met ADDM network surveillance criteria for an ASD at 8 years of age. Prevalence of ASDs was greater for non-Hispanic whites than for other racial/ethnic groups and for married than for nonmarried mothers (Table 1). Prevalence of ASDs also increased with increasing maternal age. The ASD prevalence for children born to mothers with a college degree was more than twice that compared with mothers with less than a high school education. Patterns of higher ASD prevalence for non-Hispanic whites and with higher maternal education held for some case subgroups: ADDM-determined AD, ADDM-determined ASD-NOS, and ASD without co-occurring ID. In contrast, the prevalence of ASD with co-occurring ID was almost two times as prevalent for non-Hispanic blacks compared with other racial/ethnic groups and did not exhibit a gradient across levels of maternal education (Table 1).

In our source population, 13% of all mothers smoked cigarettes during pregnancy (Table 1). The percent smoking in pregnancy was greater for mothers with lower education, younger age at her child’s birth, and not married. Non-Hispanic white mothers were more likely to smoke during pregnancy compared with non-Hispanic black mothers. The association between maternal smoking and maternal education was especially striking: Mothers with less than a high school education at the time of the child’s birth were 13 times as likely to smoke during pregnancy compared with those with a college degree.

**Figure 1:**
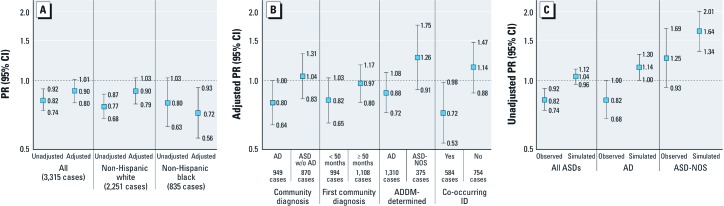
(*A*) Unadjusted and adjusted PRs (95% CI) of maternal smoking and ASD stratified by race/ethnicity. (*B*) Adjusted PRs (95% CI) of maternal smoking and ASD case subgroups. (*C*) Unadjusted PRs (95% CI) of maternal smoking in pregnancy in observed data and simulated data assuming outcome misclassification. w/o, without.

Among children recognized by ADDM as having an ASD, fewer had a report of maternal smoking in pregnancy (11%) compared with the source population (13%) (Table 2). The adjusted association between smoking and ASDs was inverse, with a lower prevalence of surveillance-ascertained ASD for women who reported smoking cigarettes in pregnancy compared with those who didn’t smoke (Figure 1A). Adjustment for maternal age, education, and marital status brought estimates upward and closer to, but still below, the null, indicating a downward confounding bias. Maternal education was the strongest confounder. Additional adjustment for county population size, birth year, surveillance site, and a cross-product between birth year and surveillance site did not alter estimates (results not shown).

Our criteria for modification of the association between maternal smoking and ASD were satisfied for race/ethnicity (likelihood ratio test *p* = 0.05): The estimated protective effect of maternal smoking on the prevalence of ASD appeared stronger for non-Hispanic blacks [PR = 0.72 (95% CI: 0.56, 0.93)] than for non-Hispanic whites [PR = 0.90 (95% CI: 0.79, 1.03)] (Figure 1A). Other potential modifiers (child’s sex, maternal education, birth-year, and county population size) did not meet modification criteria. For example, the likelihood ratio test of modification by child’s sex yielded *p* = 0.49; adjusted PRs and 95% CIs of maternal smoking in pregnancy and ASD were, for girls: 0.83 (0.64, 1.08) and for boys: 0.92 (0.81, 1.05).

We considered case subgroups defined by whether children with an ASD had other recorded characteristics or prior diagnoses that may indicate higher-functioning (such as ASD-NOS or ASD without co-occurring ID) or lower-functioning characteristics (such as AD or ASD with ID). Similarly to the inverse crude association between maternal smoking with ASDs overall, the proportion with maternal smoking in pregnancy was generally lower for these case subgroups than for the corresponding source populations (Table 2). For example, 9.0% of ADDM-determined AD reported maternal smoking compared with 10.7% in the corresponding source population. One notable exception was that children with ADDM-determined ASD-NOS (13.1%) had mothers who smoked, compared with 10.7% in the source population.

We examined adjusted associations between maternal smoking in pregnancy with each case subgroup. A consistent pattern emerged whereby lower-functioning subgroups had inverse associations with maternal smoking, whereas higher-functioning subgroups had null or positive associations (Figure 1B). Lower-functioning subgroups with below-null associations were community-diagnosed AD, earlier community diagnosis, ADDM-determined AD, and ASD with co-occurring ID. The higher-functioning subgroups with near-null associations with maternal smoking included community diagnosis of ASD without AD, a later community diagnosis, and ASD without co-occurring ID. The higher-functioning subgroup with a positive association was ADDM-determined ASD-NOS. Although the pattern of inverse point estimates for lower-functioning ASD subtypes versus positive point estimates for higher-functioning ASD subtypes held across all available subgrouping variables, it should be noted that all 95% CIs included the null and exhibited some degree of overlap with the complementary case subgroup.

*Sensitivity analysis results.* Our sensitivity analysis of differential outcome misclassification by level of maternal education yielded corrected PRs that were higher than the naive unadjusted estimates [Figure 1C; also see Supplemental Material, Tables S2 and S3 (http://ox.doi.org/10.1289/ehp.1104556)]. This upward correction held for all ASDs and the case subgroups AD and ASD-NOS as determined by ADDM clinicians, resulting in positive associations with maternal smoking across the board. The strongest association with maternal smoking was found for the higher-functioning case subgroup, ASD-NOS, with a corrected unadjusted association of 1.64 (95% CI: 1.34, 2.01).

Outcome misclassification–corrected results were affected by the assumed level of specificity (i.e., the estimated proportion of negative diagnoses that are correct). For example, the corrected result for all ASDs assuming a specificity of 0.998 was 0.94 (95% CI: 0.85, 1.04) compared with that assuming a specificity of 1.00: 1.04 (95% CI: 0.96, 1.12). We could not assume a specificity lower than 0.998 because such assumptions resulted in negative cell counts.

The subanalysis to assess the impact of removing infant deaths from the denominator used a subsample of 273,454 births. The adjusted PR limited to these areas with available data on infant mortality and retaining infant deaths was almost identical to the adjusted estimate excluding infant deaths (data not shown).

Our sensitivity analysis of a potential bias due to residential mobility, which was limited to North Carolina data, suggested no impact. The PR using the included North Carolina birth cohort as a denominator was almost identical to the PR using the subset of those children who resided within the surveillance area at age 8 years (data not shown). This result suggested that this bias due to missing cases is minimal in this region where approximately 10% migrated out of the surveillance area between birth and age 8 years.

## Discussion

Using autism surveillance and birth certificate data, we estimated the association between maternal smoking in pregnancy with ASDs identified among children 8 years of age. The primary analyses indicated a slightly inverse association with all ASDs and a suggestion that associations may differ by case subgroups. Sensitivity analyses that assumed ASD under-ascertainment varied by level of maternal education raised the possibility that observed protective associations between maternal smoking in pregnancy and ASDs were biased downward. If true, associations with maternal smoking may generally be null, but may differ by ASD subgroup.

Associations between maternal smoking and higher-functioning ASD subgroups may be positive, in contrast to those with lower-functioning ASD subgroups, which appeared null. Such findings are consistent with results from a large study using Swedish record linkages that also found higher, above-null associations with ASD without co-occurring ID, a higher functioning subgroup [PR observed to be approximately 1.13 (95% CI: 0.95, 1.25)] and inverse associations for ASD with co-occurring ID, a lower-functioning subgroup [PR approximately 0.91 (95% CI: 0.78, 1.06)] (Lee et al. 2011). Other findings that suggest more susceptibility for children with higher-functioning ASDs include a reported association between maternal smoking and scores on the Autism Spectrum Screening Questionnaire, an instrument designed to screen for symptoms of Asperger’s disorder and higher-functioning autism, among adolescents (Ehlers et al. 1999; Indredavik et al. 2007). Last, a prior U.S. study found a stronger positive association between maternal smoking in pregnancy for children with the broader classification of pervasive developmental disorder not otherwise specified compared with AD (Juul-Dam et al. 2001). Taken together, these findings raise the possibility that a higher-functioning subgroup of ASDs may be etiologically distinct and influenced by maternal smoking in pregnancy.

Our analyses suggesting that higher-functioning ASD subgroups may be caused in part by maternal smoking in pregnancy had several limitations and warrant cautious interpretation. The ASD subgroup variables were imperfect, relying on the child’s access to evaluation services and the documentation by a myriad of community providers, rather than direct clinical observation. An alternative explanation of the findings of a positive association for ADDM-determined ASD-NOS is that it is spurious, given the many subgroups examined. In addition, it is the smallest subgroup and may have incurred a larger degree of bias away from the null than the more common subgroups (Nemes et al. 2009). This higher-functioning subgroup may be more heterogeneous and so may contain children with other developmental delays, such as co-occurring attention deficit/hyperactivity disorder, known to be associated with nicotine exposure (Braun et al. 2006; Goldstein and Schwebach 2004; Simonoff et al. 2008).

Our sensitivity analysis of the impact of under-ascertainment of ASD in children with mothers of lower education indicated the possibility of a downward bias, so that associations were elevated after correction. If, as we assumed, ASDs are under-ascertained in groups with lower maternal education in ADDM data, a bias may occur because of the strong association between maternal education and smoking patterns. Because the same variable—maternal education—was used to apply weighted outcome misclassification corrections, and serves as the most important confounder, simultaneous consideration of confounding and outcome misclassification was not possible, and is a limitation of this approach. Such results must be interpreted with caution; they are contingent on the correctness of assumptions. Furthermore, the stated CIs do not account for all layers of uncertainty implicit in a sensitivity analysis, and so are too narrow to reflect the 95% coverage.

Future studies must account for the potential of under-ascertainment bias for studies of ASDs and exposures that vary with social class. Almost all environmental risk factors exhibit such gradients. We recommend that a cohort be comprehensively evaluated for ASDs or that assessment of ASD symptoms occur independently of access to services. The potential for this type of bias in studies of environmental contaminants is not limited to ASDs, but may occur for other health outcomes in which diagnosis is complicated and sensitive to demographic and societal influences.

Differences in ascertainment may help to explain discrepancies between overall null results found in these U.S. data with elevated associations found in some European studies (Hultman et al. 2002; Larsson et al. 2005; Larsson et al. 2009). The European studies took place in a context of universal access to diagnostic services, so that underascertainment may be infrequent. The social context of smoking also differs in some areas of Europe: Smoking is not as strongly associated with lower maternal education as it is in the United States, raising the possibility that a different direction of confounding bias contributed to different results between studies. Indeed, the direction of confounding by social factors in a similar large study in Sweden was upward, so that when accounted for, elevated associations were attenuated to the null (Lee et al. 2011). This is in contrast to confounding in these U.S. data, in which adjustment for social factors resulted in higher estimates.

Several explanations are consistent with the modification of the smoking-ASD association by race/ethnicity observed in these data. Residual confounding may play out differently by racial/ethnic group. Race may be serving as a marker of access to developmental evaluation services, beyond that captured by maternal education (Mandell et al. 2009). Observed modification may reflect differences in case phenotype; for example, non-Hispanic blacks were more likely to have co-occurring ID in our data compared with other racial/ethnic groups. Consequently, if associations between smoking and ASD vary by the presence of co-occurring ID, observed associations for non-Hispanic blacks will reflect a different weighting of these subgroups. Tobacco exposure–related factors may also be at play in the observed modification by race/ethnicity: Non-Hispanic black mothers smoked fewer cigarettes in these data than did non-Hispanic white mothers, and consistent evidence supports that the metabolism of tobacco smoke constituents differs by race/ethnicity (Perez-Stable et al. 1998).

Strengths of our design included a population base, large sample size, and information on autism characteristics and co-occurring conditions. These characteristics allowed us to estimate effects separately by racial/ethnic group and case subgroup with reasonable precision. Case ascertainment was standardized based on DSM-IV-TR criteria and subject to rigorous quality control procedures (Van Naarden Braun et al. 2007). Additional strengths of our design included the multiple sensitivity analyses to explore data limitations, suggesting that the impacts of infant deaths and outward migration were negligible.

Birth certificate reports of maternal smoking in pregnancy are considered to be of reasonable quality, with a 0.8 concordance with the medical record (Dietz et al. 2011). Advantages of this measure include that it occurs before the development of ASD symptoms, and so is not subject to recall bias that differs by outcome status. Limitations of our exposure measure include that it does not reflect secondhand smoke exposure, does not include postnatal exposure, and does not capture differences in cigarette composition, inhalation, or metabolism. Some studies have suggested that birth certificate underreporting of smoking may be greater with higher maternal education (Vinikoor et al. 2010). Our preliminary evaluations of the impact of differential exposure misclassification by maternal education suggested a negligible impact on findings.

## Conclusions

After correcting for expected bias due to differential under-ascertainment by maternal education, no association between maternal smoking in pregnancy and ASDs, generally, was apparent. The positive association with ASD-NOS, albeit observed in a small and heterogeneous subgroup, suggests that the higher-functioning disorders on the autism spectrum may be influenced by maternal smoking in pregnancy, and deserve further attention.

## Supplemental Material

(111 KB) PDF
