# Supplementary material for: Maternal Smoking during Pregnancy and the Prevalence of Autism Spectrum Disorders, Using Data from the Autism and Developmental Disabilities Monitoring Network
Source: Environ Health Perspect. 2012 Apr 25;120(7):1042–8. doi: 10.1289/ehp.1104556 (PMC3404663; doi:10.1289/ehp.1104556)
Supplement: (111 KB) PDF [file ehp.1104556.s001.pdf]

Supplemental Material

Maternal Smoking During Pregnancy and the Prevalence of Autism Spectrum Disorders Using Data from the Autism and Developmental Disabilities Monitoring Network

Amy E. Kalkbrenner, Joe M. Braun, Maureen S. Durkin, Matthew J. Maenner, Christopher Cunniff, Li-Ching Lee, Sydney Pettygrove, Joyce S. Nicholas, Julie L. Daniels

Contents

Supplemental Material, Table S1. Deriving assumed sensitivity of outcome classification, within level of maternal education, among those not reporting smoking in pregnancy, for Autism Spectrum Disorders, Autistic Disorder, and Autism Spectrum Disorder-Not Otherwise Specified — Page 2

Supplemental Material, Table S2. Counts of children with an Autism Spectrum Disorder, observed and simulated assuming outcome misclassification, and simulated unadjusted prevalence ratios for maternal smoking in pregnancy — Page 3

Supplemental Material, Table S3. Simulated unadjusted prevalence ratios for maternal smoking in pregnancy for case subgroups: children with Autistic Disorder and Autism Spectrum Disorder-Not Otherwise Specified, assuming outcome misclassification — Page 4

Supplemental Material, Table S1. Deriving assumed sensitivity of outcome classification, within level of maternal education, among those not reporting smoking in pregnancy, for Autism Spectrum Disorders, Autistic Disorder, and Autism Spectrum Disorder-Not Otherwise Specified

	Autism Spectrum Disorders (ASD)				Autistic Disorder (AD)				Autism Spectrum Disorders-Not Otherwise Specified (ASD-NOS)			
Maternal Education	Observed Cases (N)	Observed Prevalence per 1,000	Simulated Cases (N)[a]	Derived Sensitivity	Observed Cases (N)	Observed Prevalence per 1,000	Simulated Cases (N)[b]	Derived Sensitivity	Observed Cases (N)	Observed Prevalence per 1,000	Corrected Cases (N)[c]	Derived Sensitivity
< High school	261	3.01	740	0.35	89	2.11	303	0.29	30	0.71	89	0.34
High school degree	757	4.50	1435	0.53	285	3.67	557	0.51	74	0.95	164	0.45
Some college	771	6.14	1072	0.72	328	5.41	435	0.75	78	1.29	128	0.61
College degree	1156	6.82	1446	0.80	490	5.74	613	0.80	144	1.69	180	0.80

ASD = Autism Spectrum Disorders, AD = Autistic Disorder, ASD-NOS = Autism Spectrum Disorder-Not Otherwise Specified

Autistic Disorder and Autism Spectrum Disorder-Not Otherwise Specified were determined from developmental evaluation record review, using standard criteria, applied by clinicians from the Autism and Developmental Disabilities Monitoring Network.

a. ASD: Assuming prevalence of 8.53/1000 among non-smokers, in each strata of education, which corresponds to 80% sensitivity in strata with college degree.

b. AD: Assuming prevalence of 7.18/1000 among non-smokers, in each strata of education, which corresponds to 80% sensitivity in strata with college degree.

c. ASD-NOS: Assuming prevalence of 2.11/1000 among non-smokers, in each strata of education, which corresponds to 80% sensitivity in strata with college degree.

Supplemental Material, Table S2. Counts of children with an Autism Spectrum Disorder, observed and simulated assuming outcome misclassification, and simulated unadjusted prevalence ratios for maternal smoking in pregnancy

Maternal Education	Maternal Smoking in Pregnancy	Source Population (N)	Observed Cases (N)	Observed Prevalence per 1,000	Observed Prevalence Ratio	Sensitivity (from Table S1)	Simulated Cases (N)	Simulated Prevalence per 1,000	Simulated Prevalence Ratio
< High school	No	86,708	261	3.01	Ref	0.35	740	8.53	Ref
	Yes	30,592	109	3.56	1.18	0.35	309	10.10	1.18
High school degree	No	168,272	757	4.50	Ref	0.53	1435	8.53	Ref
	Yes	35,754	153	4.28	0.95	0.53	290	8.11	0.95
Some college	No	125,655	771	6.14	Ref	0.72	1072	8.53	Ref
	Yes	13,414	73	5.44	0.89	0.72	101	7.57	0.89
College degree	No	169,471	1156	6.82	Ref	0.80	1446	8.53	Ref
	Yes	4,123	35	8.49	1.24	0.80	44	10.62	1.24
Total	No	550,106	2,945	5.35	Ref	NA	4692	8.53	Ref
	Yes	83,883	370	4.41	0.82	NA	744	8.87	1.04

Supplemental Material, Table S3. Simulated unadjusted prevalence ratios for maternal smoking in pregnancy for case subgroups: children with Autistic Disorder and Autism Spectrum Disorder-Not Otherwise Specified, assuming outcome misclassification

			Autistic Disorder (AD)				Autism Spectrum Disorder-Not Otherwise Specified			
Maternal Education	Maternal Smoking in Pregnancy	Source Population (N)	Observed Cases (N)	Observed Prevalence Ratio	Sensitivity (from Table S1)	Simulated Prevalence Ratio	Observed Cases (N)	Observed Prevalence Ratio	Sensitivity (from Table S1)	Simulated Prevalence Ratio
< High school	No	42,150	89	Ref	0.29	Ref	30	Ref	0.34	Ref
	Yes	11,570	38	1.56	0.29	1.56	13	1.58	0.34	1.58
High school degree	No	77,556	285	Ref	0.51	Ref	74	Ref	0.45	Ref
	Yes	13,179	40	0.83	0.51	0.83	25	1.99	0.45	1.99
Some college	No	60,574	328	Ref	0.75	Ref	78	Ref	0.61	Ref
	Yes	5,386	30	1.03	0.75	1.03	7	1.01	0.61	1.01
College degree	No	85,326	490	Ref	0.80	Ref	144	Ref	0.80	Ref
	Yes	1,752	10	0.99	0.80	0.99	4	1.35	0.80	1.35
Total	No	265,606	1,192	Ref	NA	Ref	326	Ref	NA	Ref
	Yes	31,887	118	0.82	NA	1.14	49	1.25	NA	1.64
